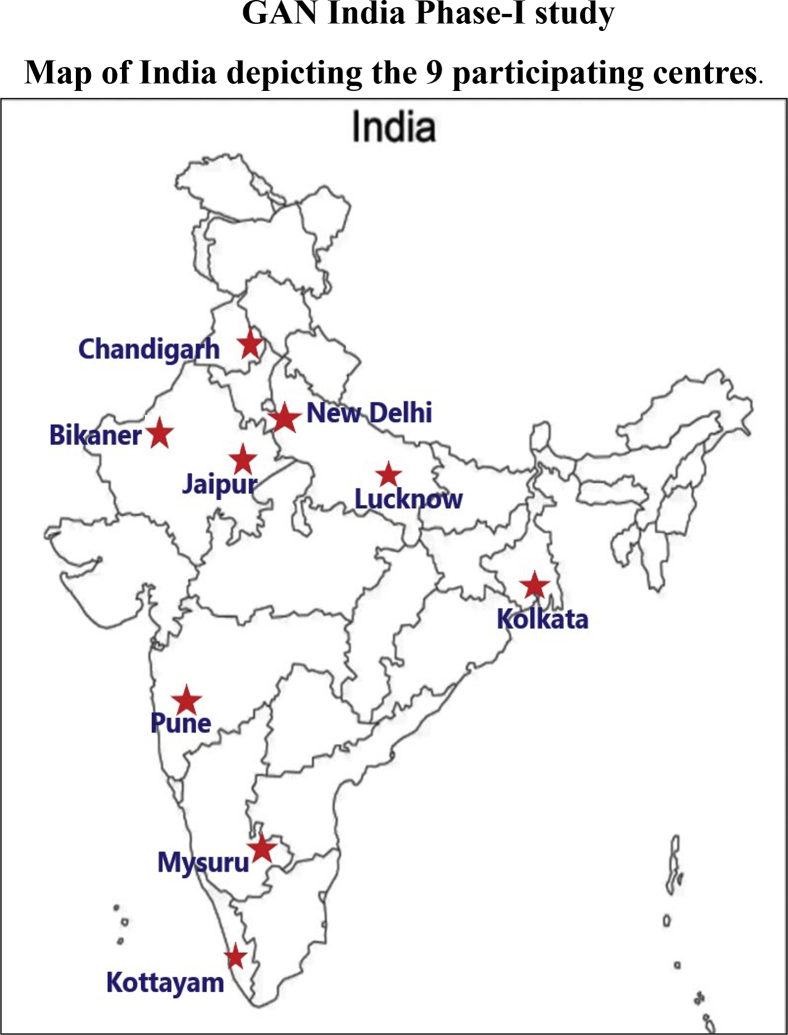# Global Asthma Network Phase I, India: Results for allergic rhinitis and eczema in 127,309 children and adults

**DOI:** 10.1016/j.jacig.2022.01.004

**Published:** 2022-03-09

**Authors:** Monica Barne, Sheetu Singh, Daya Krishan Mangal, Meenu Singh, Shally Awasthi, Padkuduru A. Mahesh, Sushil K. Kabra, Sabir Mohammed, Thevaruparambil U. Sukumaran, Aloke G. Ghoshal, Sanjeev Sinha, Sanjay K. Kochar, Nishtha Singh, Udaiveer Singh, Kamalesh Kumar Patel, Arvind Kumar Sharma, Bhushan Girase, Sapna Madas, Anil Chauhan, Niranjan Sit, Jayaraj B. Siddaiah, Virendra Singh, Sundeep Salvi

**Affiliations:** a Chest Research Foundation, Pune, India; b Pulmocare Research and Education Foundation, Pune, India; c Department of Pulmonary Medicine, Rajasthan Hospital, Jaipur, India; d Department of Public Health and Epidemiology, IIHMR University, Jaipur, India; e Department of Advanced Pediatrics, Postgraduate Institute of Medical Education & Research, Chandigarh, India; f Department of Pediatrics, King George’s Medical University, Lucknow, India; g Department of Pulmonary Medicine, Jagadguru Sri Shivarathreeshwara Medical College, JSSAHER, Mysuru, India; h Department of Pediatrics, All India Institute of Medical Sciences, New Delhi, India; i Department of Medicine, Sardar Patel Medical College, Bikaner, India; j Department of Pediatrics, Carithas Hospital, Thellakom, Kottayam, India; k Department of Respiratory Medicine, National Allergy Asthma Bronchitis Institute, Kolkata, India; l Department of Medicine, All India Institute of Medical Sciences, New Delhi, India; m Department of Respiratory Medicine, Asthma Bhawan, Jaipur, India; n Division of Research, Asthma Bhawan, Jaipur, India; o Department of Community Medicine, Mahatma Gandhi Medical College and Hospital, Jaipur, India; p PATH India, New Delhi, India

**Keywords:** Allergic rhinitis, eczema, prevalence, risk factors, India, children, adolescents, adults

## Abstract

**Background:**

The Global Asthma Network phase I study in India aimed to study the prevalence, time trends, and associated risk factors of allergic rhinitis and eczema among 6-7-year-old, and 13-14-year-old school children and their parents. **Objectives:** The objective of the study was to understand the current prevalence and associated risk factors of Allergic Rhinitis and Eczema in India among 6-7-year-olds, 13-14-year-olds and in their parents/guardians for newer directions to health care providers, policy makers and academicians.

**Methods:**

Cross-sectional, multicenter study using self- and parent-administered questionnaire among randomly selected school children aged 6 to 7 years from 8 centers and 13 to 14 years from 9 centers and their respective parents/guardians across India.

**Results:**

Prevalence of allergic rhinitis (AR) (%, 95% CI) among 20,084 6-7-year-olds (children), 25,887 13-14-year-olds (adolescents), and 81,296 adults/parents was 7.7% (7.4%-8.1%), 23.5% (23.0%-24.1%), and 9.8% (9.55%-9.96%) and that of eczema was 2.5% (2.3%-2.7%), 3.5% (3.27%-3.71%), and 9.9% (9.7%-10.1%), respectively. Among 6-7-year-olds, AR and eczema showed a significantly (*P* < .001) declining time trend since the International Study of Asthma and Allergies in school children phase III in 2002-2003. Among 13-14-year-olds, AR (*P* < .01) but not eczema showed a significant temporal decline. Coexisting atopic condition, parental history of atopy, and some environmental factors consistent with previous studies were significant risk factors among children and adolescents. AR or eczema in either parent strongly predicted the same atopic condition among their adolescent offspring. Among adults, coexisting atopic condition was the strongest predictor of either AR or eczema.

**Conclusions:**

There is a slight declining time trend of AR and eczema in India over 2 decades among children and adolescents. Nearly 10% of Indian adults suffer from AR and eczema. Although genetic factors had the strongest association for AR and eczema among all age groups, certain early-life and environmental exposures need consideration to devise preventative strategies.

Allergic rhinitis (AR) is a chronic atopic disease characterized by bouts of sneezing, runny nose, and blocked and itchy nose particularly on exposure to environmental triggers such as dust, smoke, fungi, pollen, or other known allergens.^1^ It is common in school children and adolescents, with prevalence ranging between 0.1% and 45% globally.^2^^,^^3^ When associated with ocular symptoms, it is termed allergic rhinoconjunctivitis (ARC). Eczema is an atopic skin condition that usually manifests in early childhood as a chronic itchy rash on the flexural surfaces. It is a major predictor for development of AR and asthma later in life.^4^, ^5^, ^6^ Both AR and eczema impact quality of life adversely and cause significant economic burden.^7^^,^^8^

The International Study of Asthma and Allergic Diseases in School Children (ISAAC), phase I was initiated in 1995^9^ to study the global prevalence, severity, and association with environmental risk factors of asthma, AR, and eczema using a standardized validated tool and uniform methodology. The ISAAC phase II (1998) aimed at identifying the etiological factors, and the phase III study (2001-2003) was a repetition of the phase I study to assess temporal trends in prevalence.

The ISAAC phase III studied 1.9 million children from 98 countries^10^ and showed a wide heterogeneity in the prevalence of AR and eczema globally. In India, the ISAAC phase III was conducted between 2001 and 2003 among 44,928 6-7-year-olds and 48,088 13-14-year-olds.^11^ The prevalence of AR and ARC was 11.3% (7.3%, 26.7%) and 3.9% (1.8%, 8.6%), respectively, in the 6-7-year-olds and 24.4% (4.1%, 45.7%) and 10.9% (0.9%, 23.6%) in the 13-14-year-olds, respectively. The prevalence of eczema reported was 2.8% (0.9%, 6.2%) among the 6-7-year-olds and 3.7% (0.9%, 9.2%) among the 13-14-year-olds, respectively. Risk factors associated with AR among 6-7-year-olds and 13-14-year-olds were passage of trucks near home, maternal and paternal smoking, paracetamol intake in the past 12 months, and watching television for more than 3 hours per day. In addition, paternal smoking, use of paracetamol and antibiotics during infancy, and using wood as fuel for cooking were risk factors among 6-7-year-olds, and lack of exercise was associated among 13-14-year-olds. Similar risk factors were found with eczema for both the age groups.

The ISAAC officially concluded in 2012. The Global Asthma Network (The GAN)^10^ was formed together by scientists from the ISAAC committee and the International Union Against Tuberculosis and Lung Disease (The Union). GAN continued the work that ISAAC had initiated and extended the survey to include parents of children participating in the study to bridge the data gap about prevalence of asthma, AR, and eczema in adults especially in the low- and middle-income countries.

In 2017, the GAN-India phase I study was initiated in 9 Indian cities to understand the current prevalence of asthma, AR, ARC, and eczema in 6-7-year-olds, 13-14-year-olds, and their parents/guardians. In this article, we report the prevalence, time trends, and risk factors for AR, ARC, and eczema among the 3 study groups.

## Methods

The study protocol and methodology of GAN are described in an earlier publication.^10^ In this cross-sectional, phase I GAN-India study, we repeated 7 centers that had participated in the earlier ISAAC phase III and recruited 2 new centers for better nationwide representation The GAN phase I survey was conducted among 13-14-year-olds across 9 centers in India (Bikaner, Chandigarh, Delhi, Jaipur, Kolkata, Kottayam, Mysuru, Lucknow, and Pune) and at 8 centers among 6-7-year-olds, because Kolkata did not participate in this age group. The study was coordinated by the National Data Coordinating Center, Jaipur, and was approved by ethics committees of all the respective centers. It was registered with the Clinical Trial Registry, India (CTRI/2018/02/011758), and was conducted between August 2017 and February 2018 across all the centers.

In accordance with the GAN protocol, the targeted sample size at each center was 3000 6-7-year-olds and 3000 13-14-year-olds along with their parents/guardians. This sample size gives sufficient power to detect a 5% difference between 2 centers with 99% certainty (at 1% level of significance).^10^ All 9 study centers were urban cities with varying population between 1.0 million (Chandigarh) and 16.8 million (Delhi). To achieve the sample size, a list of all schools within the predefined geographical boundary of the city was obtained from the district education authorities, with due permission. Each city was divided into 4 zones. Indian Institute of Health Management Research University (IIHMR), Jaipur, India, randomly selected an equal proportion of schools from each of the 4 zones to give us the required sample size, with some additional schools as backup in case any refused permission. Schools were sequentially approached as per the randomized list, and consenting schools were enrolled.

The research tool used was the standardized GAN questionnaire with few India-specific questions added with prior approval from the GAN committee. The questionnaire was developed in English and translated into vernacular languages (Hindi, Bengali, Marathi, Punjabi, Kannada, and Malayalam), and was validated with back-translations. English and/or vernacular questionnaires were used as per the school’s preference.

The study team included field investigators and data entry operators from all 9 centers who were trained in the study procedures and data collection methods, at IIHMR, Jaipur.

The study was conducted with due permission from the head of the school and with the help of a study coordinator and class teacher of the classrooms that contained children from the required age groups. The study was introduced as a health-related survey without mentioning the words asthma or allergy.

A passive informed consent process was implemented as per the GAN protocol. Parents were asked to telephonically contact the study team member if they wished to refuse participation. Such students and their parents were excluded from the study.

On the predesignated date and time, the study team visited the classrooms, distributed the questionnaire, and recorded the height and weight of the students. The adolescent group self-responded to their questionnaire at school if their parents had consented. They took the adult questionnaires home for their parent(s)/guardian(s) to complete. The younger age group (6-7 years) took both the child and adult questionnaires home. The parents/guardians completed the questionnaire both for their child/ward and for themselves. All questionnaires were submitted back to the respective class teacher. The study team then collected the completed questionnaire from the teacher. The parent and child questionnaires were given the same code number to study familial correlations. The study team resolved any discrepancies or missing data by contacting the parents through the school authorities.

The defining criteria for AR and eczema were as per those used in the ISAAC-Global publications^3^^,^^8^ and are given in the tables of prevalence (Tables II and III). Wheeze in the past 12 months was defined as asthma.

### Data management and statistical analysis

Coding of the data and data entry were done as per the GAN protocol. The data were entered by the respective centers, except for Kottayam, for which the entry was done by the National Data Collection Center. Ten percent of the data was double entered by IIHMR for quality checks, with a permissible mismatch of up to 2.0%. Any discrepancies beyond that were mutually resolved by the IIHMR team and the respective center. Consolidated data from all centers were sent to the GAN global center in Auckland, New Zealand, for initial quality check, and subsequently to the main data center in London, United Kingdom, where the data were rechecked. The clean data set was locked and used for analysis.

Using SPSS (Version 27, Bangalore, India), descriptive analysis and charts were used to describe demographic characteristics, prevalence rates of different symptoms, and health-related data. Continuous variables were presented using N, mean ± SD, and categorical variables were presented as frequency and percentage. Bivariate and multivariate analyses were performed to estimate the impact of environmental and health-related factors for presence of AR, ARC, and eczema.

Bivariate analysis was performed using chi-square tests for categorical variables and student *t* test for continuous variables. Results of chi-square tests were presented as odds ratios (ORs) with 95% CI. Unadjusted *P* values less than .05 were considered significant and were further included for multiple comparisons. Multivariate analysis was performed using binary logistic regression model to identify the variables associated with the presence of AR/ARC, eczema, or asthma. The results were presented using adjusted ORs and 95% CIs.

Prevalence of current AR and eczema as reported in ISAAC phase I, phase III, and GAN were compared using chi-square test. A *P* value of less than .05 was considered statistically significant.

Parent and children data sets were merged to analyze parental history as a risk factor for AR and eczema in children. The presence of current symptoms was correlated with the environmental risk factors to determine associations. Data from ISAAC phase I were extracted from the data available on the ISAAC Website, and data for phase III were extracted from the data published by Singh et al.^11^

## Results

The age-specific GAN questionnaire was administered to 23,947, 26,808, and 81,798 participants, respectively, for 6-7-year-olds, 13-14-year-olds, and parents of both age groups from across 8 centers for 6-7-year-olds and 9 centers for 13-14-year-olds (Table I).

**Table I:** GAN-India: Centerwise details of randomization, recruitment, and response rate

Center	6-7-y-olds				13-14-y-olds				Adults/parents	
	No. of primary schools randomized	No. of primary schools participated	No. of children approached	No. of children participated	No. of secondary schools randomized	No. of secondary schools participated	No. of children approached	No. of children participated	No. of parents approached	No. of parents participated
Bikaner	100	45	3,000	2,600	100	33	3,000	2,702	10,559	10,495
Chandigarh	100	57	2,473	2,473	100	54	3,000	3,000	10,394	10,386
Delhi	100	54	3,109	2,516	100	59	3,024	3,024	9,582	9,449
Jaipur	100	44	3,030	2,296	100	57	3,066	3,060	9,017	8,933
Kolkata∗	—	—	—	—	100	37	3,000	2,998	4,138	4,096
Kottayam	52	50	3,070	2,099	42	20	2,450	2,091	7,028	6,940
Lucknow	100	32	3,251	2,969	100	31	3,160	2,969	11,891	11,820
Mysore	100	30	3,003	2,730	100	29	3,067	3,051	11,181	11,178
Pune	101	26	3,011	2,404	100	34	3,041	3,030	8,008	8,000
India	753	338	23,947	20,087	842	354	26,808	25,925	81,798	81,297

∗ Kolkata did not participate in the 6-7-y-old age group.

The response rates for the 3 age groups were 84%, 97%, and 99%, respectively. Male (M) to female (F) ratio of the responses was 52:48, 49:51, and 49.8:50.2, respectively, for children, adolescents, and adults.

**Fig 1:**
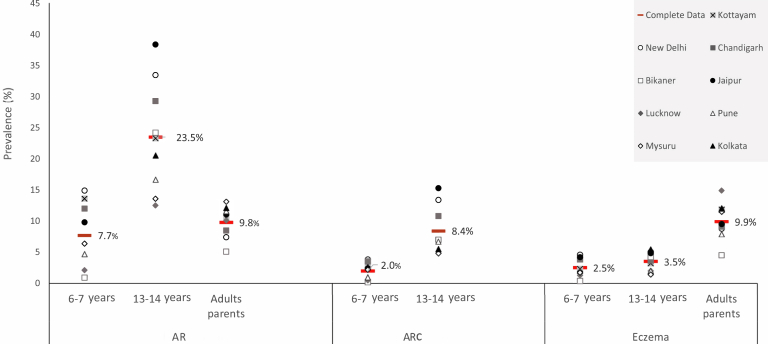
Center-specific and total prevalence of AR, ARC, and eczema among school children aged 6 to 7 years, 13 to 14 years, and their parents.

The total and centerwise prevalence of AR, ARC, and eczema for all 3 age groups is shown in Fig 1. The prevalence of AR among 6-7-year-olds was 7.7% (95% CI, 7.4%-8.1%), with a significantly higher prevalence among boys (M: 9.2% [95% CI, 8.7%-9.8%], F: 6.1% [95% CI, 5.7%-6.6%]; *P* < .0001). The prevalence of ARC was 2% (95% CI, 1.8%-2.2%) and was higher among boys (M: 2.46% [95% CI, 2.2%-2.8%], F: 1.5% [95% CI, 1.3%-1.8%]; *P* < .0001). The prevalence of AR among 13-14-year-olds was 23.5% (95% CI, 23.0%-24.1%) and that of ARC was 8.43% (95% CI, 8.1%-8.7%) with similar sex distribution. Among the adult population, the prevalence of AR was 9.8% (95% CI, 9.5%-9.9%) with no sexwise difference.

Prevalence of eczema was significantly higher among boys in both student groups, 2.5% (95% CI, 2.3%-2.7%) (M: 2.9% [95% CI, 2.55%-3.19%] vs F: 2.1% [95% CI, 1.83%-2.40%]; *P* < .001) in 6-7-year-olds and 3.48% (95% CI, 3.27%-3.71%) (M: 4.17% [95% CI, 3.86%-4.56%] vs F: 2.83% [95% CI, 2.57%-3.14%]; *P* < .0001) among 13-14-year-olds. Prevalence of eczema among adults was 9.9% (95% CI, 9.7%-10.1%) without any sex difference.

There was a wide variation in the prevalence of AR, ARC, and eczema between different centers among all the 3 age groups.

**Fig 2:**
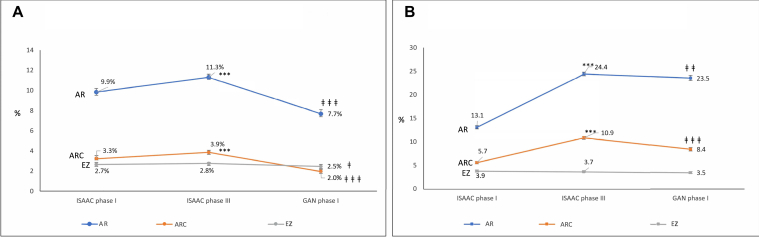
Time trends in prevalence of AR, ARC, and eczema in the 6-7-year-olds (**A**) and 13-14-year-olds (**B**) across ISAAC phase I, ISAAC phase III, and GAN phase I. ∗*P* < .05, ∗∗*P* < .01, ∗∗∗*P* < .001 when ISAAC phase I compared with ISAAC phase Ill. ‡*P* < .05, ‡‡*P* < .01, ‡‡‡*P* < .001 when ISAAC phase Ill compared with GAN phase I.

Time trends in AR, ARC, and eczema from ISAAC-1, ISAAC-3, and GAN are depicted in Fig 2 and show significant reduction in all prevalence rates (*P* < .05). Eczema in adolescents did not show a statistically significant decline.

Table II presents details of labeled disease, morbidity, and severity for AR and ARC. About 0.9% of the 6-7-year-olds had moderately severe AR and 0.3% had severe AR. Significantly more girls had severe AR as compared with boys (M: 0.18%, F: 0.38%, *P* = .007). Among adolescents, 1.3% and 0.6% had moderately severe and severe AR, respectively, without any sex difference.

**Table II:** Prevalence of self-reported, labeled and doctor diagnosed condition, prevalence of ARC, moderately severe and severe AR

Symptoms	6-7 y (N = 20,084)				13-14 y (N = 25,925)				Adults (N = 81,297)∗			
	Male (N = 10,441)	Female (N = 9,643)	*P* value	Total	Male (N = 12,671)	Female (N = 13,216)	*P* value	Total	Male (N = 40,468)	Female (N = 40,815)	*P* value	Total
Presence of nasal symptoms ever	1,453 (13.92)	1,057 (10.96)	<.0001	2,510 (12.5)	4,212 (32.79)	4,155 (31.44)	.02	8,367 (32.30)	NA	NA	NA	NA
Nasal symptoms in past 12 mo	961 (9.20)	593 (6.10)	<.0001	1,554 (7.70)	3,025 (23.87)	3,071 (23.24)	.232	6,096 (23.50)	NA	NA	NA	NA
Labeled AR	847 (8.11)	611 (6.15)	<.0001	1,458 (7.30)	1,722 (13.59)	1,772 (13.41)	.672	3,494 (13.50)	3,912 (9.69)	4,018 (9.89)	.562	7,930 (9.80)
Diagnosis confirmed by doctor	521 (4.99)	370 (3.84)	<.0001	891 (4.40)	798 (6.30)	801 (6.06)	.423	1,599 (6.20)	2,212 (5.70)	2,340 (5.50)	.315	4,552 (5.60)
ARC	257 (2.46)	145 (1.50)	<.0001	402 (2.00)	1,057 (8.33)	1,128 (8.52)	.582	2,185 (8.43)	NA	NA	NA	NA
Nose problem accompanied by itchy nose	348 (3.33)	192 (1.99)	<.0001	540 (2.70)	804 (6.34)	853 (6.45)	.718	1,667 (6.40)	NA	NA	NA	NA
Moderately severe AR	119 (1.14)	64 (0.66)	.0003	183 (0.90)	156 (1.23)	183 (1.38)	.288	339 (1.30)	NA	NA	NA	NA
Severe AR	19 (0.18)	37 (0.38)	.007	56 (0.30)	73 (0.58)	88 (0.66)	.413	161 (0.60)	NA	NA	NA	NA

*NA*, Questions not included in adult questionnaire.

Values are n (%).

AR, Presence of nasal symptoms in the past 12 mo.

ARC, (Presence of nasal symptoms in the past 12 mo + nose problem been accompanied by itchy watery eyes in past 12 mo)/Total number of completed questionnaires.

Moderately severe ARC, condition interfering with daily activities a moderate amount.

Severe ARC, condition interfering with daily activities a lot.

∗ Sex is not defined for 13 adults.

Labeled disease condition, severity, and morbidity for eczema are presented in Table III. Moderately severe eczema and severe eczema prevalence was 1.1% and 0.3%, respectively, in children and 2.89% and 1% among adolescents. About 2.2% of children and 3.9% of adolescents reported never being free of symptoms in the past 12 months, indicating high morbidity.

**Table III:** Prevalence of eczema, labeled ever and doctor-confirmed eczema and severity in children and adults

Symptoms	6-7 y (N = 20,084)				13-14 y (N = 25,887)				Adults (N = 81,296)∗			
	M (N = 10,441)	F (N = 9,643)	*P* value	Total	M (N = 12,671)	F (N = 13,216)	*P* value	Total	M (N = 40,468)	F (N = 40,815)	*P* value	Total
Prevalence of symptoms ever	605 (5.79)	403 (4.18)	<.0001	1,008 (5.01)	1,309 (10.33)	1,080 (8.17)	<.0001	2,469 (9.50)	NA	NA	NA	NA
Prevalence of current symptoms	298 (2.85)	202 (2.09)	<.0001	498 (2.47)	532 (4.17)	376 (2.83)	<.0001	902 (3.48)	NA	NA	NA	NA
Labeled disease condition	558 (5.34)	563 (5.84)	.038	1,121 (5.60)	1,390 (10.97)	996 (7.54)	<.0001	2,386 (9.20)	4,121 (10.20)	3,963 (9.70)	.191	8,084 (9.90)
Diagnosis confirmed by doctor	351 (3.36)	324 (3.36)	.01	675 (3.40)	570 (4.50)	394 (2.98)	<.0001	964 (3.70)	2,375 (5.90)	2,381 (5.80)	.025	4,757 (5.90)
Moderately severe eczema	131 (1.25)	91 (0.94)	.035	222 (1.10)	553 (4.36)	449 (3.40)	.0001	1,002 (3.90)	NA	NA	NA	NA
Severe eczema	47 (0.45)	20 (0.21)	.003	67 (0.30)	366 (2.89)	280 (2.12)	.0001	646 (2.50)	NA	NA	NA	NA

*NA*, Questions not included in adult questionnaire.

Values are n (%).

Eczema, Itchy rash at any time in the past 12 mo + rash on flexural surfaces.

Moderately severe eczema, condition interfering with daily activities a moderate amount.

Severe eczema, condition interfering with daily activities a lot.

High morbidity due to eczema, rash not cleared at any time in the past 12 mo.

∗ Sex is not defined for 13 adults.

### Associations

**Fig 3:**
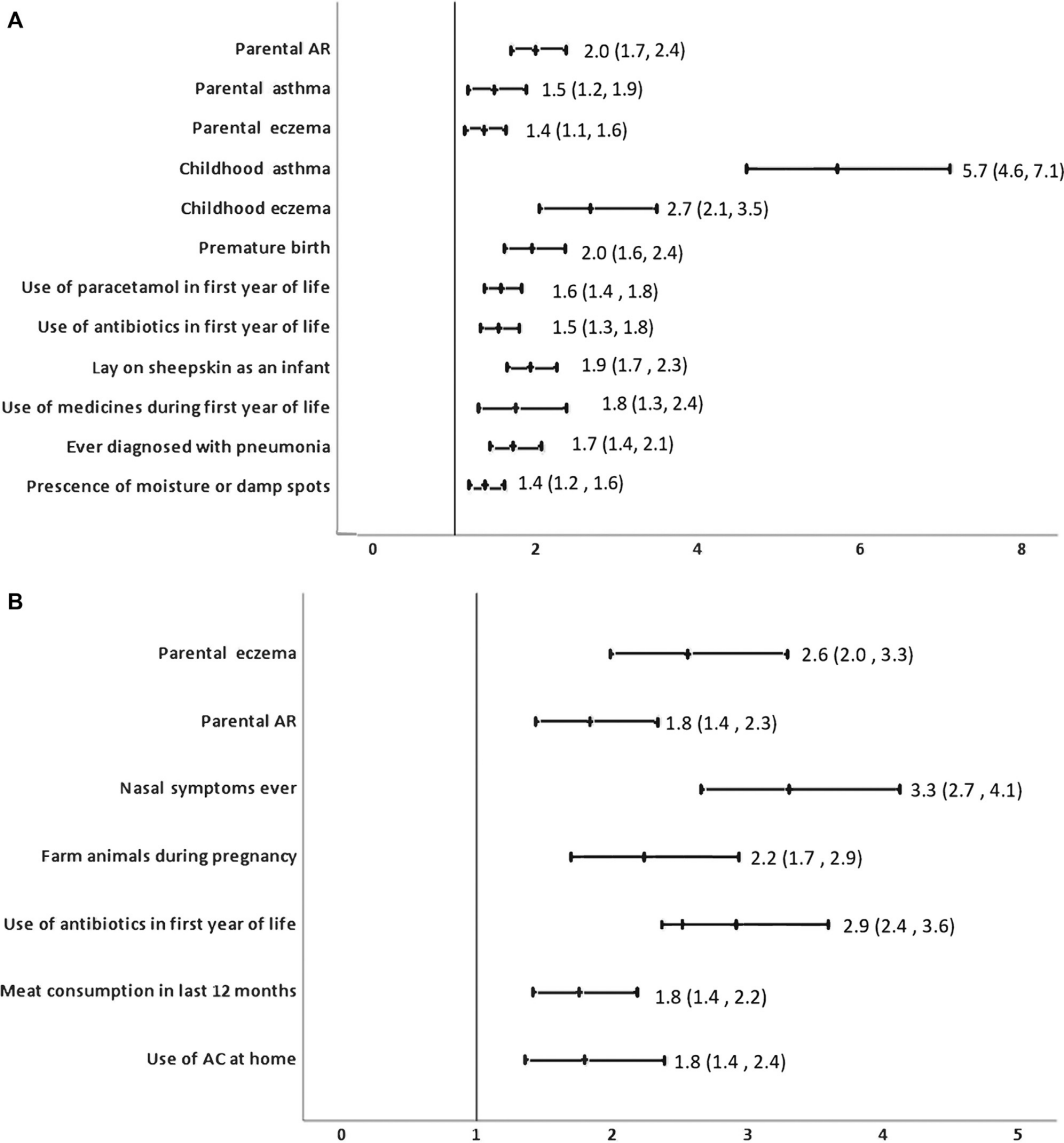
Risk factors associated with AR and eczema among 6-7-year-olds showing adjusted OR and 95% CI. *AC*, Air-conditioner.

**Fig 4:**
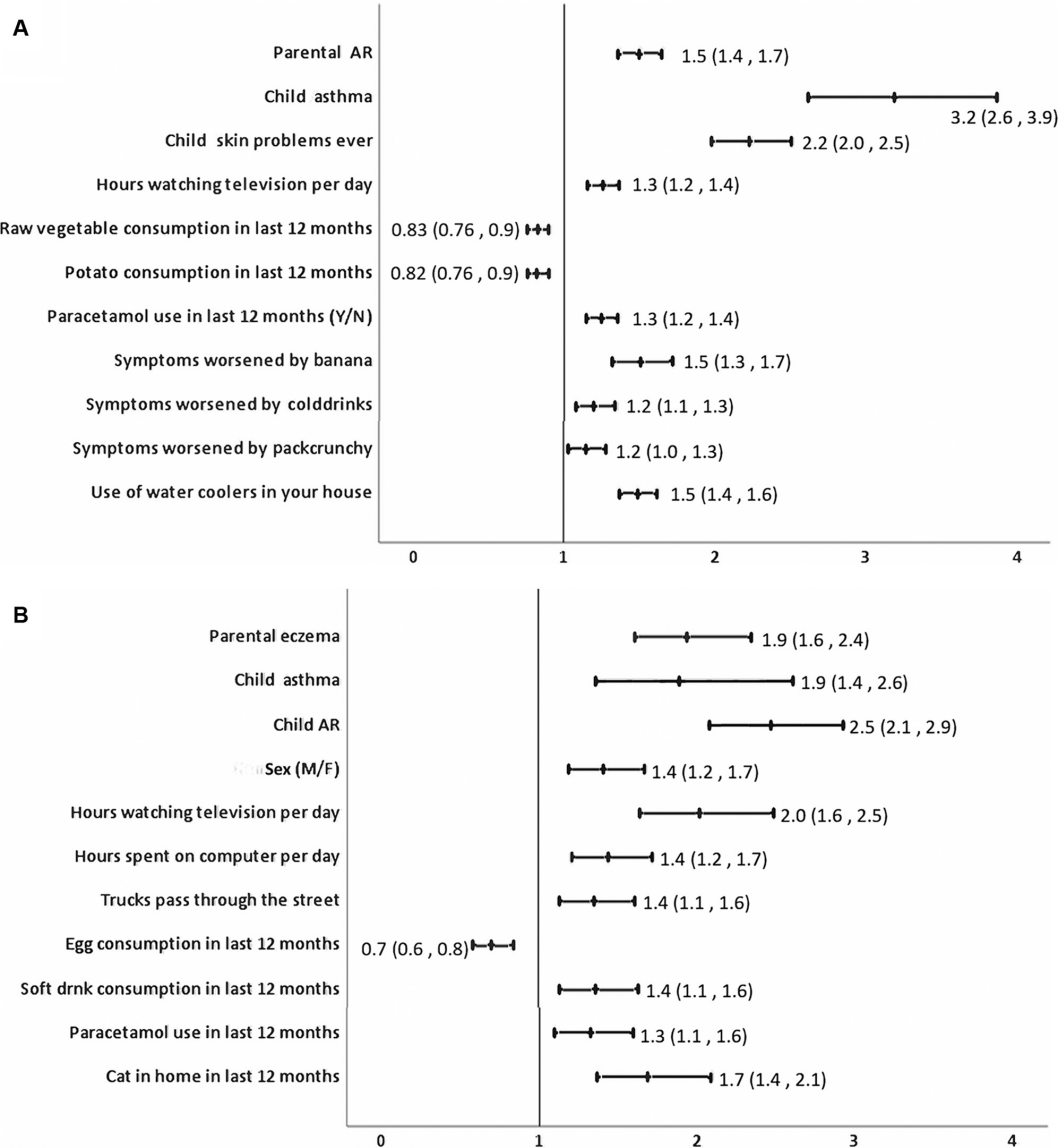
Risk factors for AR and eczema for 13-14-year-olds showing adjusted ORs and 95% CI.

**Fig 5:**
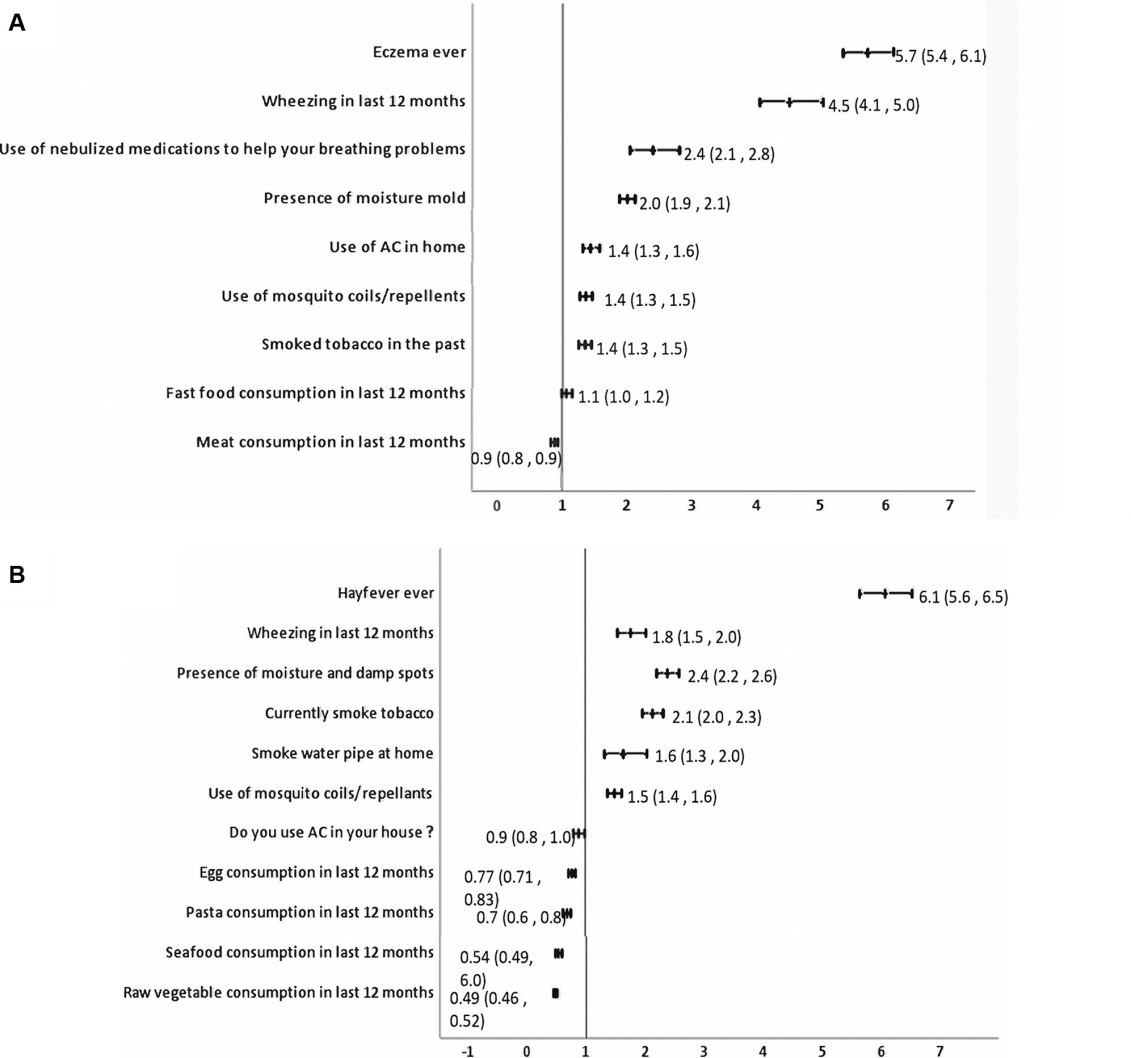
Risk factors for AR and eczema in adults showing adjusted ORs and 95% CI.

**Fig 6:**
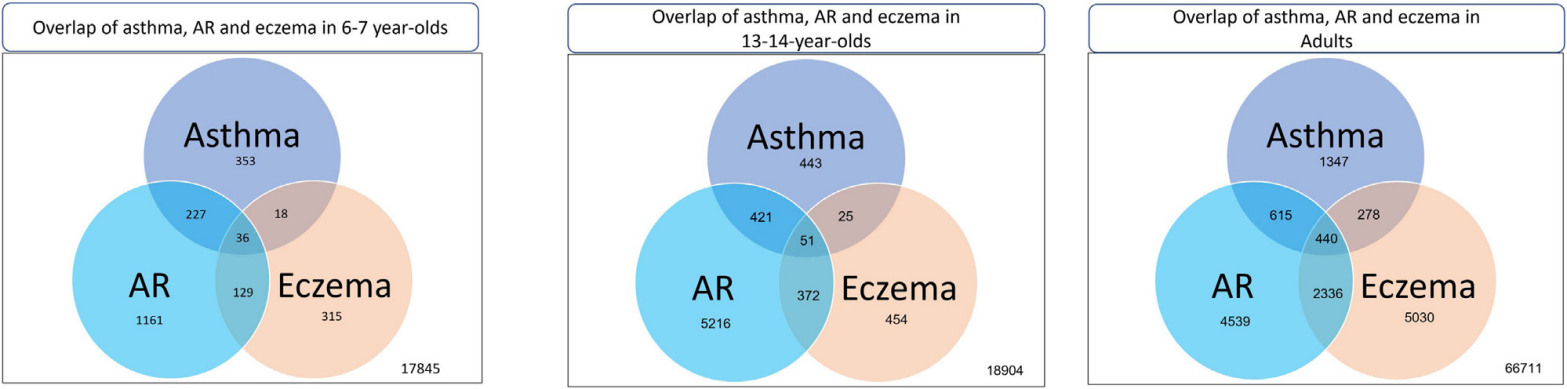
Overlap of asthma, AR, and eczema in the 3 age groups.

Significant associations with any coexisting atopic conditions, parental history of atopy, and environmental factors for AR and eczema with adjusted OR are given in Figs 3 (*A* and *B*), 4 (*A* and *B*), and 5 (*A* and *B*) for children, adolescents, and adults, respectively. Complete analysis of the associations with unadjusted and adjusted OR and factors that were protective is given in Online Repository data available at www.jaci-global.org. Fig 6 depicts the Venn diagram of the overlapping conditions in all the 3 age groups.

#### Associations for AR

Among 6-7-year-olds, risk of AR significantly increased with presence of coexisting asthma and eczema. Risk of AR was significantly high if either parent had asthma or AR or eczema. Among adolescents, AR was associated significantly with coexisting asthma and skin problems ever but not with eczema. Parental AR was positively associated with AR in adolescents. Among adults, AR significantly increased with coexisting asthma and eczema. Nongenetic risk factors for AR among 6-7-year-olds were premature birth, laying on sheepskin during infancy, consumption of medicines, paracetamol, or antibiotics during infancy, ever had pneumonia, and presence of moisture or damp spots in house. Among adolescents, AR was associated with increase in symptoms after consumption of banana, watching television for more than 1 hour per day, and use of paracetamol during the past 12 months.

Among adults, AR was significantly associated with use of nebulized medications in the past 12 months, presence of moisture or molds in the house, use of air-conditioner at home, use of mosquito coils/repellants, smoking tobacco ever, and fast-food consumption in the past 12 months.

#### Associations for eczema

Frequent nasal symptoms, presence of parental AR, or eczema in either parent was significantly associated with eczema among 6-7-year-olds. Among 13-14-year-olds, eczema was significantly associated both with coexisting asthma and with AR. Presence of parental eczema was significantly associated with the 13-14-year-olds having eczema. Among adults, eczema was associated with presence of coexisting asthma and AR. Environmental associations for eczema among 6-7-year-olds were use of antibiotics during infancy, intrauterine exposure to farm animals, use of air-conditioner at home, and meat consumption during the past 12 months. Among adolescents, eczema was significantly associated with more than 1 hour of watching television per day, presence of cat in the home during the past 12 months, more than 1 hour spent on computer per day, consumption of soft drinks in last 12 months, passage of heavy trucks through the street, and current use of paracetamol. Environmental factors for eczema among adults included presence of moisture and damp walls in the house, current use of tobacco, smoking water pipe at home, and use of mosquito coils and repellants or incense sticks at home.

## Discussion

The GAN phase I study explored the prevalence of AR and eczema among 127,309 children, adolescents, and their parents/guardians among 9 geographically spread cities and is one of the largest studies reported in India during the last 2 decades.

The prevalence of AR in India among 6-7-year-olds and 13-14-year-olds was 7.7% and 23.5%, respectively. The findings from the GAN study for the Bangkok region have shown a prevalence of 38.2% and 48.8%, respectively, for these age groups.^12^ GAN results from the Yazd province report a prevalence of 36.3% among 13-14-year-olds.^13^ GAN results from other Asian countries are awaited.

The ISAAC phase III conducted in 2002-2003 had shown a significant increase in prevalence of AR and ARC among the 6-7-year-olds and the 13-14-year-olds (*P* < .001) in comparison to ISAAC phase I. GAN phase I done almost 2 decades after ISAAC phase III has shown a significant decline in comparison to the ISAAC phase III. These time trends are consistent with the findings from global data and that from Asian countries as per findings of GAN phase I for AR for both children and adolescents reported recently.^14^ Despite the decrease, we observe that nearly a quarter of the adolescent school children in India suffer from AR and 8.5% have ARC. This calls for urgent attention. A significant proportion of those who had allergic rhinitis (74%) did not even receive a diagnosis of AR by their doctor. We therefore believe that there is an urgent need to create awareness about this huge problem in the community, reduce the level of nondiagnosis and underdiagnosis through appropriate health care capacity building and infrastructure, and treat them effectively because this disease has a huge impact on the quality of life. Susceptible individuals with a strong family history could be advised preventive methods such as environment control and trigger avoidance. Compared with ISAAC phase III results, our results show a significant reduction in the prevalence of eczema among the 6-7-year-olds. The slight decrease in eczema in adolescents, though not statistically significant, is consistent with findings of GAN phase I studies in Iran^13^ and Ecquador.^15^ However, global trends are awaited.

The prevalence of AR among 81,297 adults in India from across 9 different cities was 9.5%. To our knowledge, this is the largest study for AR prevalence among Indian adults. A previous large-scale study for asthma prevalence among 73,605 adults in 4 cities in India had reported a prevalence of recurrent coryza between 1.4% and 7.6%, and recurrent skin rashes between 0.3% and 3.9%.^16^ A smaller yet significant community-based study of 1203 adults in Delhi in 2012 showed 11% prevalence for AR,^17^ and our study substantiates this finding. There is dearth of data on community prevalence of eczema among adults in India,^18^ and ours is the first study to report a high prevalence of 9.9%. Among the Asian countries, a study conducted between 2012 and 2013 among 2482 adults reports that prevalence of self-reported AR was 4.5%, 9.7%, and 8.6% in Ukraine, Kazakhstan, and Azerbaijan, respectively.^19^ Studies from the last couple of decades have found varied prevalence in the Asian countries from 6.1% in China,^20^ 20.4% in Kuwait,^21^ 25% in UAE,^22^ 34% in Thailand,^23^ and 53% in Malaysia.^24^ Prevalence of eczema in various Asian countries shows a prevalence of 9.2% in Kuwait.^21^ Earlier studies have shown prevalence of 9.4% in Bangkok in 2002^25^ and 15% in 2007.^26^

Wide heterogeneity prevails in the prevalence of AR between different centers among children (0.9%-14.9%), adolescents (12.5%-38.4%), and adults (5.1%-14.9%) across India. Eczema also varied between 0.8% and 6% for children, 1.4% and 5.4% for adolescents, and 4.5% and 14.9% for adults. This heterogeneity among the centers is expected because India is a very large country with wide variation in geography, climate, genetics, and sociocultural and dietary practices, which may influence the prevalence.

Strongest risk factors for AR among 6-7-year-olds were coexisting asthma and eczema with a 5.7-fold and 2.6-fold increased risk, respectively, reiterating that asthma, AR, and eczema are part of a single disease complex and often coexist.^27^, ^28^, ^29^ This strong association of asthma but not eczema persists during adolescence though adolescents with AR did report a 2.2-fold higher prevalence of skin problems ever. Coexisting conditions were a strong predictor for AR in adults, with a 4.5-fold increase with asthma and a 5.7-fold increase with eczema. This is consistent with existing literature.^27^^,^^29^^,^^30^ Our study also showed that AR was associated with a 2.4-fold higher use of nebulized medications in the past 12 months among adults, indicating poor asthma control, probably attributable to the AR. Inadequately managed AR leads to recurrent asthma exacerbations, increasing health care burden and costs.

Among children, AR was strongly associated with all 3 atopic conditions in parents but among adolescents, only parental AR increased the risk by 50%. For eczema in children, parental eczema and parental AR were a risk factor but among adolescents, it was only parental eczema but not asthma or AR that shared an increased association with eczema by 94%. This indicates that parental AR or eczema is a strong predictor of whether their adolescent children will develop AR or eczema, and ours is probably the first study to show this.

Previous studies have reported premature births as a protective factor for AR,^31^, ^32^, ^33^ but our study found that premature birth increased the risk for AR by 96% in concordance with the findings of Mitselou et al^34^ who reported a hazard ratio of 1.12 (95% CI, 1.04-1.20) for AR in preterm children.^34^ Association of premature birth with asthma is known,^35^^,^^36^ but our study establishes high risk for AR as well. Laying on sheep skin in infancy, a significant high-risk factor for AR, probably indicates use of sheep-wool blankets in urban population. As compared with ISAAC phase III, use of paracetamol and antibiotics in infancy retained their association in our study but exposure to maternal smoking, air pollution, and sedentary lifestyle were not found significant when adjusted for parental history and coexisting atopy, indicating that genetic predisposition and early-life exposures are more significant predictors of AR in children.

Among adolescents, consumption of banana was the most significant nongenetic association for AR. This was an India-specific question that substantiates practicing clinicians’ observations of the role of banana, as a common trigger in India. Another India-specific question about the presence of water-coolers in the house showed increased association with AR among adolescents. This may possibly be due to fungus growing in the improperly maintained water-cooler. Use of paracetamol in the past 12 months and watching television were risk factors consistent with the ISAAC phase III observations.

Among adults, India-specific questions added on indoor air pollutants such as mosquito coils, incense sticks, and mosquito repellants yielded significant association and are modifiable factors providing scope for intervention.

Increased risk of eczema among 6-7-year-olds associated with use of antibiotics during infancy is consistent with existing literature^37^^,^^38^ and ISAAC phase III data from India. Intrauterine exposure to farm animals showed greater odds (OR, 2.24) than global findings from ISAAC phase III (OR, 1.38).^39^ Association with air-conditioner in the house may either be a risk factor or a preference by the parents for the child’s skin condition as reported earlier.^40^ Meat consumption was a risk factor consistent with earlier findings.^41^ Among adolescents, sedentary lifestyle spent indoors, heavy truck-traffic passage through the street, and consumption of paracetamol in the past year were risk factors consistent with ISAAC phase III for eczema. Cats in the house were also found significantly associated for eczema. Among adults, risk factors for eczema were like those for AR with damp walls, smoking tobacco, and exposure to indoor air pollutants and are largely preventable.

### Strengths of our study

The use of a standardized protocol and questionnaire, 3 different age groups, large sample size spatially distributed across the geography, and addition of India-specific questions are some of the key strengths. Availability of parent and child data allowed us newer insights into the role of genetics and predictors of AR and eczema, which was not possible in the studies that were part of ISAAC.

### Limitations

The study was limited to urban cities only. This was a self-reported, questionnaire-based study and lack of standard terminology in colloquial languages for terms such as “hay fever” and “eczema” may possibly be a limitation despite our best efforts to validate the translations.

### Conclusions

The prevalence of AR and eczema in India has declined among the 6-7-year-olds and 13-14-year-olds over the past 2 decades. Yet, about a quarter of the adolescent population in India suffers from AR. This is indeed alarming and needs attention. Adolescence is a vulnerable age, and presence of an adverse health condition can significantly affect overall development. Parental AR or eczema is a strong predictor of AR or eczema, respectively, among adolescents. India has a very large population of adults who suffer from AR and eczema, which needs to be diagnosed and managed appropriately. Family history and early-life exposures are strong risk factors for AR and eczema in children.

**Clinical implications**

In India, high prevalence of AR among adolescents and high prevalence of AR and eczema among adults with strong familial associations need due consideration for early diagnosis and proper management.

## Figures and Tables

**Fig E1:**