# Supplementary material for: Global Asthma Network Phase I, India: Results for allergic rhinitis and eczema in 127,309 children and adults
Source: J Allergy Clin Immunol Glob. 2022 Mar 9;1(2):51–60. doi: 10.1016/j.jacig.2022.01.004 (PMC10509898; doi:10.1016/j.jacig.2022.01.004)
Supplement: Supplementary Material [file mmc1.docx]

# **Title: Global Asthma Network Phase-I, India: Results for allergic rhinitis and eczema in 127,309 children and adults**

**SUPPLEMENTAL DATA**

**GAN Study : Association and Logistic Regression analysis result tables:**

**Table 1a: Complete results of regression analysis for allergic rhinitis (AR) for children 6-7 year old.**

| **Risk Factors** | **Unadjusted OR**  **(95% CI)** | **p-value** | **Adjusted OR**  **(95% CI)** | **p-value** |
| --- | --- | --- | --- | --- |
| Parental AR | 3.77 (3.34, 4.24) | <0.001 | 2.01 (1.70, 2.38) | <0.001 |
| Parental Asthma | 3.52 (2.97, 4.19) | <0.001 | 1.49 (1.17, 1.89) | 0.022 |
| Parental Eczema | 2.67 (2.34, 3.04) | <0.001 | 1.37 (1.13, 1.64) | <0.001 |
| Childhood Asthma | 9.79 (8.27, 11.60) | <0.001 | 5.73 (4.61, 7.12) | <0.001 |
| Childhood Eczema | 6.54 (5.38, 7.95) | <0.001 | 2.68 (2.05, 3.50) | <0.001 |
| Premature birth | 3.16 (2.75, 3.63) | <0.001 | 1.96 (1.62, 2.37) | <0.001 |
| Use of Paracetamol in first year of life | 2.45 (2.19, 2.74) | <0.001 | 1.58 (1.37, 1.83) | <0.001 |
| Use of antibiotics in first year of life | 3.42 (3.05, 3.84) | <0.001 | 1.54 (1.32, 1.80) | <0.001 |
| Lay on sheepskin as an infant | 3.83 (3.40, 4.32) | <0.001 | 1.94 (1.65, 2.27) | <0.001 |
| Use of medicines during first year of life | 7.03 (5.76, 8.59) | <0.001 | 1.76 (1.30, 2.39) | <0.001 |
| Ever diagnosed with pneumonia | 3.56 (3.12, 4.07) | <0.001 | 1.73 (1.44, 2.08) | <0.001 |
| Prescence of moisture or damp spots | 2.56 (2.27, 2.88) | <0.001 | 1.38 (1.18, 1.62) | <0.001 |
| Use Mosquito coils/Repellents | 0.66 (0.58, 0.74) | <0.001 |  |  |
| Gender | 1.55 (1.39, 1.72) | <0.001 |  |  |
| Paracetamol use in last 12 months (once per year / once per month Vs. Never) | 2.07 (1.83, 2.36) | <0.001 |  |  |
| Farm animals during pregnancy | 2.89 (2.48, 3.36) | <0.001 |  |  |
| Mother smoked during pregnancy | 5.10 (3.78, 6.89) | <0.001 |  |  |
| Breastfeeding duration (less than 6 months Vs. More than 6 months VS) | 1.36 (1.17, 1.58) | <0.001 |  |  |
| Exclusive breastfeeding duration (Less than 4 months Vs. More than 4 months) | 1.13 (0.99, 1.29) | 0.071 |  |  |
| Chest infections in first year of life | 1.89 (1.70, 2.12) | <0.001 |  |  |
| Cat in the home during first year of life | 2.75 (2.32, 3.26) | <0.001 |  |  |
| Dog in the home during first year of life | 2.29 (1.93, 2.72) | <0.001 |  |  |
| Farm animals during first year of life | 4.25 ( 3.65, 4.95) | <0.001 |  |  |
| Wheezing during first year of life | 7.53 (6.36, 8.91) | <0.001 |  |  |
| Out of home care younger than 3 years | 1.89 (1.67, 2.13) | <0.001 |  |  |
| Out of home care older than 3 years | 1.18 (1.05, 1.32) | 0.004 |  |  |
| Hours per week of physical activity (Once or twice per week + 3 or more per week vs. Never or occasionally) | 0.34 (0.29, 0.41) | <0.001 |  |  |
| Hours watching television per day (More than 1 hr. Vs. less than 1 hr) | 1.27 (1.13, 1.42) | <0.001 |  |  |
| Hours spent on computer per day | 0.64 (0.56, 0.74) | <0.001 |  |  |
| Twin | 1.85 (1.36, 2.50) | <0.001 |  |  |
| Made changes in home to prevent symptoms | 3.10 (2.63, 3.66) | <0.001 |  |  |
| Meat consumption in last 12 months (once or twice per week / most or all days vs. never or occasionally) | 0.86 (0.76, 0.97) | 0.017 |  |  |
| Fruit consumption in last 12 months | 0.43 (0.39, 0.49) | <0.001 |  |  |
| Cooked vegetable consumption in last 12 months | 0.49 (0.43, 0.55) | <0.001 |  |  |
| Raw vegetable consumption in last 12 months | 0.45 (0.40, 0.50) | <0.001 |  |  |
| Pulse consumption in last 12 months | 0.46 (0.41, 0.53) | <0.001 |  |  |
| Cereal consumption in last 12 months | 0.55 (0.48, 0.63) | <0.001 |  |  |
| Bread consumption in last 12 months | 0.46 (0.41, 0.52) | <0.001 |  |  |
| Pasta consumption in last 12 months | 0.93 (0.81, 1.08) | 0.362 |  |  |
| Rice consumption in last 12 months | 0.56 (0.49, 0.64) | <0.001 |  |  |
| Margarine consumption in last 12 months | 1.18 (1.02, 1.37) | 0.027 |  |  |
| Butter consumption in last 12 months | 0.77 (0.68, 0.87) | <0.001 |  |  |
| Milk consumption in last 12 months | 0.46 (0.41, 0.52) | <0.001 |  |  |
| Other dairy consumption in last 12 months | 0.50 (0.45, 0.56) | <0.001 |  |  |
| Egg consumption in last 12 months | 1.24 (1.10, 1.38) | <0.001 |  |  |
| Nut consumption in last 12 months | 0.46 (0.41, 0.51) | <0.001 |  |  |
| Potato consumption in last 12 months | 0.53 (0.47, 0.59) | <0.001 |  |  |
| Sugar consumption in last 12 months | 0.76 (0.68, 0.86) | <0.001 |  |  |
| Burger consumption in last 12 months | 0.73 (0.63, 0.83) | <0.001 |  |  |
| Fast food consumption in last 12 months | 0.93 (0.81, 1.08) | 0.342 |  |  |
| Soft drink consumption in last 12 months | 0.84 (0.73, 0.97) | 0.015 |  |  |
| Cat in home in last 12 months | 1.64 (1.33, 2.00) | <0.001 |  |  |
| Dog in home in last 12 months | 2.05 (1.72, 2.44) | <0.001 |  |  |
| Child Skin problems ever (RASHEV) | 5.44 (4.68, 6.33) | <0.001 |  |  |
| Prescence of mould spots | 2.89 (2.52, 3.29) | <0.001 |  |  |
| Prescence of moisture , mould damp spot in any room | 1.85 (1.64, 2.07) | <0.001 |  |  |
| Prescence of moisture/mould (Yes to any one of 26 & 27) | 2.34 (2.09, 2.61) | <0.001 |  |  |
| Child born by Caesarean section | 1.52 (1.35, 1.72) | <0.001 |  |  |
| Use of water coolers in your house | 0.74 (0.66, 0.82) | <0.001 |  |  |

# **Table 1(b): Complete results of regression analysis for eczema for children 6-7 year old**

| **Risk Factors** | **Unadjusted OR**  **(95% CI)** | **p-value** | **Adjusted OR**  **(95% CI)** | **p-value** |
| --- | --- | --- | --- | --- |
| Parental Eczema | 4.29 (3.53, 5.22) | <0.001 | 2.56 (1.99, 3.30) | <0.001 |
| Parental AR | 4.22 (3.49, 5.12) | <0.001 | 1.84 (1.44, 2.34) | <0.001 |
| Nasal Symptoms ever | 5.91 (4.91, 7.10) | <0.001 | 3.31 (2.66, 4.13) | <0.001 |
| Farm animals during pregnancy | 3.10 (2.44, 3.94) | <0.001 | 2.24 (1.70, 2.94) | <0.001 |
| Use of antibiotics in first year of life | 4.20 (3.48, 5.07) | <0.001 | 2.92 (2.37, 3.60) | <0.001 |
| Meat consumption in last 12 months | 1.53 (1.27, 1.85) | <0.001 | 1.76 (1.42, 2.19) | <0.001 |
| Use of AC at home | 2.0 (1.56, 2.56) | <0.001 | 1.80 (1.36, 2.39) | <0.001 |
| Use Mosquito coils/Repellents | 0.78 (0.63, 0.96) | 0.018 | 0.67 (0.53, 0.85) | <0.001 |
| Paracetamol use in last 12 months | 2.05 (1.65, 2.55) | <0.001 |  |  |
| Gender | 1.39 (1.16, 1.67) | <0.00 |  |  |
| Mother smoked during pregnancy | 4.28 (2.64, 6.93) | <0.001 |  |  |
| Child born prematurely | 3.10 (2.49, 3.87) | <0.001 |  |  |
| Paracetamol in first year of life | 2.56 (2.12, 3.13) | <0.001 |  |  |
| Paracetamol during pregnancy | 0.60 (0.49, 0.74) | <0.001 |  |  |
| Chest infections in first year of life | 2.01 (1.67, 2.42) | <0.001 |  |  |
| Lay on sheepskin as an infant | 3.35 (2.74, 4.09) | <0.001 |  |  |
| Cat in the home during first year of life | 3.57 (2.76, 4.61) | <0.001 |  |  |
| Dog in the home during first year of life | 2.63 (2.01, 3.45) | <0.001 |  |  |
| Farm animals during first year of life | 2.69 (2.04, 3.53) | <0.001 |  |  |
| Wheezing during first year of life | 6.51 (5.04, 8.41) | <0.001 |  |  |
| Medicines during first year of life | 8.01 (6.06, 10.57) | <0.001 |  |  |
| Out of home care younger than 3 years | 2.46 (2.01, 2.99) | <0.001 |  |  |
| Out of home care older than 3 years | 1.10 (0.91, 1.33) | 0.319 |  |  |
| Hours per week of physical activity | 0.51 (0.39, 0.67) | <0.001 |  |  |
| Hours watching television per day | 1.59 (1.31, 1.93) | <0.001 |  |  |
| Hours spent on computer per day | 0.49 (0.39, 0.62) | <0.001 |  |  |
| Truck pass through street (Seldom/ frequently/almost all day vs. never) | 0.80 (0.66, 0.96) | 0.015 |  |  |
| Ever diagnosed with pneumonia | 2.68 (2.13, 3.37) | <0.001 |  |  |
| Twin | 2.01 (1.24, 3.27) | 0.004 |  |  |
| Made changes in home to prevent symptoms | 3.97 (3.06, 5.14) | <0.001 |  |  |
| Fruit consumption in last 12 months | 0.53 (0.44, 0.65) | <0.001 |  |  |
| Cooked vegetable consumption in last 12 months | 0.83 (0.65, 1.06) | 0.126 |  |  |
| Raw vegetable consumption in last 12 months | 0.40 (0.33, 0.49) | <0.001 |  |  |
| Pulse consumption in last 12 months | 0.48 (0.39, 0.59) | <0.001 |  |  |
| Cereal consumption in last 12 months | 0.81 (0.64, 1.03) | 0.079 |  |  |
| Bread consumption in last 12 months | 0.77 (0.64, 0.93) | 0.007 |  |  |
| Pasta consumption in last 12 months | 0.94 (0.74, 1.19) | 0.581 |  |  |
| Rice consumption in last 12 months | 0.67 (0.54, 0.84) | <0.001 |  |  |
| Butter consumption in last 12 months | 0.77 (0.62, 0.95) | 0.013 |  |  |
| Milk consumption in last 12 months | 0.67 (0.54, 0.83) | <0.001 |  |  |
| Other dairy consumption in last 12 months | 0.60 (0.49, 0.73) | <0.001 |  |  |
| Nut consumption in last 12 months | 0.48 (0.40, 0.58) | <0.001 |  |  |
| Potato consumption in last 12 months | 0.62 (0.50, 0.76) | <0.001 |  |  |
| Sugar consumption in last 12 months | 0.86 (0.71, 1.05) | 0.134 |  |  |
| Burger consumption in last 12 months | 0.62 (0.49, 0.79) | <0.001 |  |  |
| Fast food consumption in last 12 months | 0.79 (0.62, 1.02) | 0.07 |  |  |
| Cat in home in last 12 months | 2.13 (1.58, 2.88) | <0.001 |  |  |
| Dog in home in last 12 months | 2.15 (1.63, 2.84) | <0.001 |  |  |
| Asthma (WHEZ12) | 3.94 (2.93, 5.29) | <0.001 |  |  |
| AR (Allergic Rhinitis)(PNOSE12) | 6.54 (5.38, 7.95) | <0.001 |  |  |
| Presence of Asthma (Father or Mother) | 3.58 (2.74, 4.68) | <0.001 |  |  |
| Prescence of moisture or damp spots | 2.72 (2.23, 3.13) | <0.001 |  |  |
| Presence of mould and spots parents | 2.78 (2.23, 3.46) | <0.001 |  |  |
| Prescence of moisture in any room | 1.86 (1.52, 2.27) | <0.001 |  |  |
| Presence of moisture / mold | 2.15 (1.79, 2.60) | <0.001 |  |  |
| Child born by Caesarean section | 1.51 (1.22, 1.86) | <0.001 |  |  |

**Table 2a: Complete results of regression analysis for AR for children 13-14 year old**

| **Association between Allergic Rhinitis (AR) Vs. Background Characteristics for 13-14 Years.** | **Unadjusted OR (95% CI)** | **p-value** | **Adjusted OR (95% CI)** | **p-value** |
| --- | --- | --- | --- | --- |
| Parental AR | 1.92 (1.78, 2.08) | <0.001 | 1.50 (1.36, 1.65) | <0.001 |
| Child Asthma | 3.65 (3.19, 4.17) | 0.001 | 3.19 (2.62, 3.87) | <0.001 |
| Child skin problem ever | 2.61 (2.39, 2.85) | <0.001 | 2.23 (1.98, 2.51) | <0.001 |
| Hours watching television per day | 1.35 (1.27, 1.43) | <0.001 | 1.26 (1.16, 1.37) | <0.001 |
| Raw vegetable consumption in last 12 months | 0.70 (0.66, 0.72) | <0.001 | 0.83 (0.76, 0.90) | <0.001 |
| Potato consumption in last 12 months | 0.77 (0.72, 0.83) | <0.001 | 0.82 (0.76, 0.90) | <0.001 |
| Paracetamol use in last 12 months (Y/N) | 1.39 (1.31, 1.48) | <0.001 | 1.25 (1.15, 1.36) | <0.001 |
| Symptoms worsen by Banana | 1.47 (1.34, 1.69) | <0.001 | 1.51 (1.32, 1.72) | <0.001 |
| Symptoms worsen by Cold drinks | 1.37 (1.26, 1.48) | <0.001 | 1.20 (1.08, 1.34) | <0.001 |
| Symptoms worsen by Packcrunchy | 1.29 (1.19, 1.40) | <0.001 | 1.15 (1.03, 1.28) | 0.010 |
| Use of water coolers in your house | 1.41 (1.32, 1.50) | <0.001 | 1.49 (1.37, 1.62) | <0.001 |
| Hours per week of physical activity | 1.10 (1.03, 1.18) | 0.004 |  |  |
| Hours spent on computer per day | 1.10 (1.03, 1.17) | 0.003 |  |  |
| Trucks pass through the street | 1.10 (1.04, 1.16) | 0.002 |  |  |
| Meat consumption in last 12 months | 0.82 (0.77, 0.87) | 0.000 |  |  |
| Seafood consumption in last 12 months | 0.81 (0.75, 0.87) | <0.001 |  |  |
| Fruit consumption in last 12 months | 0.87 (0.81, 0.93) | 0.001 |  |  |
| Cooked vegetable consumption in last 12 months | 0.77 (0.72, 0.82) | <0.001 |  |  |
| Pulse consumption in last 12 months | 0.80 (0.75, 0.85) | <0.001 |  |  |
| Cereal consumption in last 12 months | 0.88 (0.82, 0.93) | <0.001 |  |  |
| Bread consumption in last 12 months | 0.75 (0.71, 0.80) | <0.001 |  |  |
| Pasta consumption in last 12 months | 0.87 (0.82, 0.93) | <0.001 |  |  |
| Rice consumption in last 12 months | 0.86 (0.80, 0.92) | <0.001 |  |  |
| Margarine consumption in last 12 months | 0.80 (0.74, 0.87) | <0.001 |  |  |
| Butter consumption in last 12 months | 0.86 (0.81, 0.92) | <0.001 |  |  |
| Olive oil consumption in last 12 months | 0.88 (0.82, 0.95) | <0.001 |  |  |
| Milk consumption in last 12 months | 0.85 (0.80, 0.90) | <0.00 |  |  |
| Egg consumption in last 12 months | 0.86 (0.80, 0.91) | <0.001 |  |  |
| Nut consumption in last 12 months | 0.84 (0.79, 0.89) | <0.001 |  |  |
| Sugar consumption in last 12 months | 0.91 (0.86, 0.97) | 0.003 |  |  |
| Burger consumption in last 12 months | 0.88 (0.82, 0.94) | <0.001 |  |  |
| Fast food consumption in last 12 months | 0.80 (0.75, 0.86) | <0.001 |  |  |
| Soft drink consumption in last 12 months | 0.78 (0.73, 0.83) | <0.001 |  |  |
| Cat in home in last 12 months | 1.19 (1.09, 1.29) | <0.001 |  |  |
| Dog in home in last 12 months | 1.20 (1.11, 1.30) | <0.001 |  |  |
| Smoke water pipe at home | 1.30 (1.04, 1.62) | 0.022 |  |  |
| Use of nebulized medications to help your breathing problem | 2.04 (1.71, 2.44) | <0.001 |  |  |
| Symptoms worsen by Curdyog | 1.30 (1.18, 1.42) | <0.001 |  |  |
| Symptoms worsen by Ice-creams | 1.19 (1.12, 1.28) | <0.001 |  |  |
| Symptoms worsen by Cake pastries | 1.27 (1.13, 1.42) | <0.001 |  |  |
| Child Eczema (RASH12+SITESEV) | 3.08 (2.69, 3.53) | <0.001 |  |  |
| Parental Asthma | 1.80 (1.61, 2.02) | <0.001 |  |  |
| Parental Eczema | 1.62 (1.50, 1.75) | <0.001 |  |  |
| Prescence of moisture or damp spots | 1.48 (1.38, 1.59) | <0.001 |  |  |
| Prescence of mould spots | 1.58 (1.45, 1.71) | <0.001 |  |  |
| Prescence of moisture , mould damp spot in any room | 1.29 (1.20, 1.39) | <0.001 |  |  |
| Prescence of moisture/mould | 1.37 (1.28, 1.46) | <0.001 |  |  |
| Child born by Caesarean section | 0.89 (0.82, 0.97) | 0.005 |  |  |

**Table 2b: Complete results of regression analysis for eczema for children 13-14 year old**

| **Risk Factors** | **Unadjusted OR (95% CI)** | **p-value** | **Adjusted OR**  **(95% CI)** | **P-value** |
| --- | --- | --- | --- | --- |
| Parental Eczema | 1.86 (1.59, 2.19) | <0.001 | 1.94 (1.61, 2.35) | <0.001 |
| Child Asthma | 2.66 (2.08, 3.39) | <0.001 | 1.89 (1.36, 2.61) | <0.001 |
| Child AR | 3.08 (2.69, 3.53) | <0.001 | 2.47 (2.08, 2.93) | <0.001 |
| Gender (M/F) | 1.49 (1.30, 1.70) | <0.001 | 1.41 (1.19, 1.67) | <0.001 |
| Hours watching television per day | 2.52 (2.15, 2.96) | <0.001 | 2.02 (1.64, 2.49) | <0.001 |
| Hours spent on computer per day | 1.89 (1.65, 2.17) | <0.001 | 1.44 (1.21, 1.72) | <0.001 |
| Trucks pass through the street | 1.51 (1.32, 1.74) | <0.001 | 1.35 (1.13, 1.61) | <0.001 |
| Egg consumption in last 12 months | 0.83 (0.72, 0.95) | 0.009 | 0.70 (0.58, 0.84) | <0.001 |
| Soft drink consumption in last 12 months | 1.25 (1.09, 1.45) | 0.002 | 1.36 (1.13 (1.63) | <0.001 |
| Paracetamol use in last 12 months | 1.85 (1.59, 2.16) | <0.001 | 1.33 (1.10, 1.60) | 0.003 |
| Cat in home in last 12 months | 1.84 (1.56, 2.17) | <0.001 | 1.69 (1.37, 2.09) | <0.001 |
| Use Mosquito coils/Repellents | 0.75 (0.64, 0.87) | <0.001 | 0.66 (0.55, 0.79) | <0.001 |
| Hours per week of physical activity | 1.17 (1.00, 1.37) | 0.046 |  |  |
| Seafood consumption in last 12 months | 0.80 (0.67, 0.95) | 0.01 |  |  |
| Bread consumption in last 12 months | 1.20 (1.05, 1.38) | 0.009 |  |  |
| Pasta consumption in last 12 months | 1.24 (1.07, 1.44) | 0.004 |  |  |
| Rice consumption in last 12 months | 1.29 (1.20, 1.51) | 0.002 |  |  |
| Margarine consumption in last 12 months | 0.66 (0.54, 0.80) | <0.001 |  |  |
| Potato consumption in last 12 months | 1.22 (1.05, 1.41) | 0.008 |  |  |
| Sugar consumption in last 12 months | 1.34 (1.16, 1.55) | <0.001 |  |  |
| Dog in home in last 12 months | 1.44 (1.23, 1.70) | <0.001 |  |  |
| Smoked tobacco in the past | 1.45 (1.01, 2.07) | 0.042 |  |  |
| Smoke water pipe at home | 1.97 (1.31, 2.94) | <0.001 |  |  |
| Use of nebulized medications to help your breathing problem | 2.28 (1.65, 3.16) | <0.001 |  |  |
| Symptoms worsen by Banana | 1.28 (1.05, 1.57) | 0.015 |  |  |
| Symptoms worsen by Curdyog | 1.32 (1.08, 1.61) | 0.007 |  |  |
| Symptoms worsen by Cold drinks | 1.74 (1.48, 2.05) | <0.001 |  |  |
| Symptoms worsen by Ice-creams | 1.23 (1.05, 1.43) | 0.009 |  |  |
| Symptoms worsen by Cake pastries | 1.29 (1.01, 1.65) | 0.045 |  |  |
| Symptoms worsen by Packcrunchy | 1.36 (1.14, 1.62) | <0.001 |  |  |
| Nasal Symptoms ever (PNOSEEV) | 2.69 (2.35, 3.09) | <0.001 |  |  |
| Parental Asthma | 1.37 (1.05, 1.77) | 0.018 |  |  |
| Parental AR | 1.62 (1.36, 1.92) | <0.001 |  |  |
| Prescence of moisture or damp spots | 1.48 (1.26, 1.74) | <0.001 |  |  |
| Prescence of mould spots | 1.54 (1.29, 1.85) | <0.001 |  |  |
| Prescence of moisture , mould damp spot in any room | 1.27 (1.07, 1.50) | 0.005 |  |  |
| Prescence of moisture/mould | 1.29 (1.11, 1.50) | <0.001 |  |  |

**Table 3a: Complete results of regression analysis of AR among adults:**

| **Risk Factors** | **Unadjusted OR**  **(95% CI)** | **p-value** | **Adjusted OR**  **(95% CI)** | **p-value** |
| --- | --- | --- | --- | --- |
| Eczema ever | 7.01 (6.64, 7.40) | 0.000 | 5.73 (5.35, 6.13) | 0.000 |
| Wheezing in last 12 months | 7.16 (6.59, 7.77) | 0.000 | 4.52 (4.06, 5.04) | <0.001 |
| Use of nebulized medications to help your breathing problems | 4.88 (4.34, 5.49) | <0.001 | 2.41 (2.05, 2.82) | <0.001 |
| Presence of moisture mould | 2.97 (2.83, 3.12) | 0.000 | 2.01 (1.89, 2.13) | <0.001 |
| Use of AC in home | 1.51 (1.40, 1.63) | <0.001 | 1.44 (1.32, 1.58) | <0.001 |
| Use of mosquito coils/repellents | 1.58 (1.50, 1.68) | <0.001 | 1.37 (1.28, 1.47) | <0.001 |
| Smoked tobacco in the past | 1.99 (1.87, 2.12) | <0.001 | 1.36 (1.26, 1.46) | <0.001 |
| Fast food consumption in last 12 months | 0.83 (0.78, 0.89) | <0.001 | 1.07 (1.00, 1.16) | <0.001 |
| Meat consumption in last 12 months | 0.79 (0.75, 0.83) | <0.001 | 0.89 (0.83, 0.94) | <0.001 |
| Gender (M/F) | 0.98 (0.94, 1.03) | 0.375 |  |  |
| Presence of moisture and damp spots | 3.38 (3.21, 3.56) | 0.000 |  |  |
| Presence of mould spots | 3.41 (3.21, 3.62) | 0.000 |  |  |
| Presence of moisture_damp_mould spots in any room | 2.72 (2.57, 2.87) | <0.001 |  |  |
| Cooking fuel: Not food cooked at home | 2.06 (1.71, 2.48) | <0.001 |  |  |
| Cooking fuel: Electricity | 1.33 (1.22, 1.45) | <0.001 |  |  |
| Cooking fuel: Liquefied petroleum gas | 0.82 (0.76, 0.88) | <0.001 |  |  |
| Cooking fuel: natural gas | 1.43 (1.32, 1.56) | <0.001 |  |  |
| Cooking fuel: Biogas | 1.40 (1.24, 1.58) | <0.001 |  |  |
| Cooking fuel: Kerosene | 1.68 (1.51, 1.87) | <0.001 |  |  |
| Cooking fuel: Coal/lignite | 2.28 (1.95, 2.66) | <0.001 |  |  |
| Cooking fuel: Charcoal | 1.74 (1.42, 2.13) | <0.001 |  |  |
| Cooking fuel: Wood | 1.57 (1.45, 1.69) | <0.001 |  |  |
| Cooking fuel: Straw/shrubs/grass | 2.82 (2.43, 3.26) | <0.001 |  |  |
| Cooking fuel: Animal Dung | 2.48 (2.20, 2.80) | <0.001 |  |  |
| Cooking fuel: Agricultural crop residue | 2.63 (2.23, 3.11) | <0.001 |  |  |
| Hood | 1.25 (1.09, 1.42) | <0.001 |  |  |
| Chimney | 1.68 (1.56, 1.82) | <0.001 |  |  |
| Smoke water pipe at home | 1.93 (1.62, 2.30) | <0.001 |  |  |
| Seafood consumption in last 12 months | 0.70 (0.66, 0.74) | <0.001 |  |  |
| Fruit consumption in last 12 months | 0.84 (0.82, 0.89) | <0.001 |  |  |
| Cooked vegetable consumption in last 12 months | 0.87 (0.82, 0.92) | <0.001 |  |  |
| Raw vegetable consumption in last 12 months | 0.60 (0.57, 0.63) | <0.001 |  |  |
| Pulse consumption in last 12 months | 0.72 (0.68, 0.76) | <0.001 |  |  |
| Cereal consumption in last 12 months | 0.89 (0.84, 0.94) | <0.001 |  |  |
| Bread consumption in last 12 months | 0.56 (0.54, 0.59) | <0.001 |  |  |
| Pasta consumption in last 12 months | 0.64 (0.60, 0.68) | <0.001 |  |  |
| Rice consumption in last 12 months | 0.65 (0.61, 0.68) | <0.001 |  |  |
| Margarine consumption in last 12 months | 0.74 (0.69, 0.79) | <0.001 |  |  |
| Butter consumption in last 12 months | 0.90 (0.86, 0.95) | <0.001 |  |  |
| Olive oil consumption in last 12 months | 0.72 (0.68, 0.77) | <0.001 |  |  |
| Milk consumption in last 12 months | 0.75 (0.71, 0.79) | <0.001 |  |  |
| Other dairy consumption in last 12 months | 0.70 ( 0.66, 0.73) | <0.001 |  |  |
| Egg consumption in last 12 months | 0.74 (0.70, 0.78) | <0.001 |  |  |
| Nut consumption in last 12 months | 0.67 (0.64, 0.71) | <0.001 |  |  |
| Potato consumption in last 12 months | 0.82 (0.78, 0.87) | <0.001 |  |  |
| Sugar consumption in last 12 months | 0.84 (0.80, 0.86) | <0.001 |  |  |
| Burger consumption in last 12 months | 0.66 (0.62, 0.70) | <0.001 |  |  |
| Soft drink consumption in last 12 months | 0.83 (0.78, 0.88) | <0.001 |  |  |
| Currently smoke tobacco | 1.95 (1.85, 2.08) | <0.001 |  |  |
| Use of water cooler in home | 1.49 (1.42, 1.56) | <0.001 |  |  |

**Table 3b: Complete results of regression analysis for eczema among adults**

| **Risk Factors** | **Unadjusted OR**  **(95% CI)** | **p-value** | **Adjusted OR (95% CI)** | **p-value** |
| --- | --- | --- | --- | --- |
| Hay fever ever (AR) | 7.01 (6.64, 7.40) | <0.001 | 6.07 (5.64, 6.53) | 0.000 |
| Wheezing in last 12 months | 3.67 (3.36, 4.01) | <0.001 | 1.76 (1.54, 2.02) | <0.001 |
| Presence of moisture and damp spots | 4.72 (4.49, 4.97) | <0.001 | 2.38 (2.20, 2.58) | <0.001 |
| Currently smoke tobacco | 2.52 (2.37, 2.67) | <0.001 | 2.13 (1.96, 2.31) | <0.001 |
| Smoke water pipe at home | 2.96 (2.53, 3.46) | <0.001 | 1.64 (1.32, 2.04) | <0.001 |
| Use of Mosquito coils/Repellents | 1.89 (1.78, 2.00) | <0.001 | 1.49 (1.37, 1.61) | <0.001 |
| Use of AC at home | 1.01 (0.93, 1.09) | 0.869 | 0.89 (0.80, 0.99) | 0.012 |
| Egg consumption in last 12 months | 0.47 (0.44, 0.49) | <0.001 | 0.77 (0.71, 0.83) | <0.001 |
| Pasta consumption in last 12 months | 0.45 (0.42, 0.48) | <0.001 | 0.69 (0.62, 0.75) | <0.001 |
| Seafood consumption in last 12 months | 0.41 (0.38, 0.44) | <0.001 | 0.54 (0.49, 0.60) | <0.001 |
| Raw vegetable consumption in last 12 months | 0.35 (0.33, 0.37) | <0.001 | 0.49 (0.46, 0.52) | <0.001 |
| Sex of the respondent | 1.05 (1.01, 1.10) | 0.024 |  |  |
| Presence of mould spots | 3.69 (3.48, 3.91) | <0.001 |  |  |
| Presence of moisture_damp_mould spots in any room | 4.01 (3.78, 4.26) | <0.001 |  |  |
| Presence of moisture mold | 3.86 (3.68, 4.05) | <0.001 |  |  |
| Cooking fuel: Not food cooked at home | 2.18 (1.80, 2.65) | <0.001 |  |  |
| Cooking fuel: Electricity | 1.61 (1.47, 1.76) | <0.001 |  |  |
| Cooking fuel: natural gas | 1.50 (1.38, 1.64) | <0.001 |  |  |
| Cooking fuel: Biogas | 1.48 (1.30, 1.68) | <0.001 |  |  |
| Cooking fuel: Kerosene | 2.56 (2.32, 2.83) | <0.001 |  |  |
| Cooking fuel: Coal/lignite | 3.57 (3.09, 4.13) | <0.001 |  |  |
| Cooking fuel: Charcoal | 2.07 (1.68, 2.55) | <0.001 |  |  |
| Cooking fuel: Wood | 2.0 (1.85, 2.15) | <0.001 |  |  |
| Cooking fuel: Straw/shrubs/grass | 4.15 (3.60, 4.79) | <0.001 |  |  |
| Cooking fuel: Animal Dung | 3.87 (3.45, 4.35) | <0.001 |  |  |
| Cooking fuel: Agricultural crop residue | 3.74 (3.18, 4.40) | <0.001 |  |  |
| Use of nebulized medications to help your breathing problems | 1.39 (1.29, 1.50) | <0.001 |  |  |
| Meat consumption in last 12 months | 0.61 (0.57, 0.64) | <0.001 |  |  |
| Fruit consumption in last 12 months | 0.56 (0.53, 0.58) | <0.001 |  |  |
| Cooked vegetable consumption in last 12 months | 0.77 (0.72, 0.81) | <0.001 |  |  |
| Pulse consumption in last 12 months | 0.59 (0.56, 0.62) | <0.001 |  |  |
| Cereal consumption in last 12 months | 0.76 (0.72, 0.80) | <0.001 |  |  |
| Bread consumption in last 12 months | 0.51 (0.48, 0.54) | <0.001 |  |  |
| Rice consumption in last 12 months | 0.57 (0.54, 0.60) | <0.001 |  |  |
| Margarine consumption in last 12 months | 0.50 (0.46, 0.54) | <0.001 |  |  |
| Butter consumption in last 12 months | 0.54 (0.51, 0.57) | <0.001 |  |  |
| Olive oil consumption in last 12 months | 0.49 (0.46, 0.53) | <0.001 |  |  |
| Milk consumption in last 12 months | 0.63 (0.60, 0.66) | <0.001 |  |  |
| Other dairy consumption in last 12 months | 0.52 (0.50, 0.55) | <0.001 |  |  |
| Nut consumption in last 12 months | 0.44 (0.42, 0.47) | <0.001 |  |  |
| Potato consumption in last 12 months | 0.76 (0.73, 0.80) | <0.001 |  |  |
| Sugar consumption in last 12 months | 0.68 (0.65, 0.72) | <0.001 |  |  |
| Burger consumption in last 12 months | 0.37 (0.35, 0.40) | <0.001 |  |  |
| Fast food consumption in last 12 months | 0.43 (0.39, 0.46) | <0.001 |  |  |
| Soft drink consumption in last 12 months | 0.45 (0.42, 0.48) | <0.001 |  |  |
| Smoked tobacco in the past | 2.45 (2.34, 2.62) | <0.001 |  |  |
| Use of cooler at home | 2.57 (2.45, 2.71) | <0.001 |  |  |
